# *In Silico* Meta-Analysis of Boundary Conditions for Experimental Tests on the Lumbar Spine

**DOI:** 10.1007/s10439-022-03015-x

**Published:** 2022-07-29

**Authors:** Simone Borrelli, Giovanni Putame, Giulia Pascoletti, Mara Terzini, Elisabetta M. Zanetti

**Affiliations:** 1 Department of Mechanical and Aerospace Engineering (DIMEAS), Politecnico di Torino, C.so Duca degli Abruzzi 24, 10129 Turin, Italy; 2 PolitoBIOMedLab, Politecnico di Torino, Turin, Italy; 3 Department of Engineering, University of Perugia, Via G. Duranti 92, 06125 Perugia, Italy

**Keywords:** Lumbar spine, Biomechanics, Multibody, ROM, Follower load, Mechanical tests

## Abstract

The study of the spine range of motion under given external load has been the object of many studies in literature, finalised to a better understanding of the spine biomechanics, its physiology, eventual pathologic conditions and possible rehabilitation strategies. However, the huge amount of experimental work performed so far cannot be straightforwardly analysed due to significant differences among loading set-ups. This work performs a meta-analysis of various boundary conditions in literature, focusing on the flexion/extension behaviour of the lumbar spine. The comparison among range of motions is performed virtually through a validated multibody model. Results clearly illustrated the effect of various boundary conditions which can be met in literature, so justifying differences of biomechanical behaviours reported by authors implementing different set-up: for example, a higher value of the follower load can indeed result in a stiffer behaviour; the application of force producing spurious moments results in an apparently more deformable behaviour, however the respective effects change at various segments along the spine due to its natural curvature. These outcomes are reported not only in qualitative, but also in quantitative terms. The numerical approach here followed to perform the meta-analysis is original and it proved to be effective thanks to the bypass of the natural variability among specimens which might completely or partially hinder the effect of some boundary conditions. In addition, it can provide very complete information since the behaviour of each functional spinal unit can be recorded. On the whole, the work provided an extensive review of lumbar spine loading in flexion/extension.

**Supplementary Information:**

The online version contains supplementary material available at 10.1007/s10439-022-03015-x.

## Introduction

The biomechanics of the spine is complex because it is actually a multi-body structure, including 33 vertebrae; 9 vertebrae are actually fused (the coccyx and the sacrum), while the remaining 24 vertebrae are articulated to one another through 3 joints: one disc joint and 2 facet joints. The global deformability of the spine is so resulting from the sum of many contributions and its experimental characterization has been the object of numerous studies in literature, where the first studies are dated 1980 and since then an exponential growth has been recorded.^21^ The reasons for such a wide experimental effort are mainly the incidence of clinical problems affecting the spine such as reduced mobility, pain and other pathologies. Some studies, for example, were aimed to establish the loss of mobility which might come as a consequence of vertebral fusions^20^; other studies were addressed to define specifications for prosthetic implants^28,31^ or to simulate the outcome of surgery^18^. Zhang *et al*.^35^ focused on the characterization of the mechanical joint behavior in numerical models of the spine. The study of spine deformability at each articulation can be very helpful to discriminate between physiologic and pathologic conditions such as spondylolisthesis or disc degeneration^19^, and it can provide key information about the role of physiologic restraint structures^32^.

Given the huge amount of information that can be found in literature, obtaining the respective synthetic view is not trivial due to the variety of set-ups which have been implemented, as detailed by some very complete reviews. This paper retraces these reviews, focusing on those referring to the lumbar spine and its flexion/extension behavior. Adopting a different perspective, it is aimed to obtain a quantitative estimate of the impact of boundary conditions on the recorded mechanical behavior, as reported on the so-called ‘Range Of Motions (ROM) curves’. The final purpose is not only to establish the weight to be given to the creation of a proper experimental set-up, but also building a tool which allows to critically evaluate findings from many authors who have collected a huge amount of scientific data that deserve to be reconsidered and discussed, also considering performing further *ex vivo* tests on human specimens. The approach followed for this sort of ‘biomechanical review’ is original, since the meta-analysis is based on the employment of a multibody model where different boundary conditions can be compared on the same exact specimen, eliminating any form of experimental variability. The findings so obtained will be discussed, considering differences in experimental results among authors who made use of different experimental set-ups.

## Synthetic View of Loading Set-Ups in Literature

The literature research was performed in Medline with the following sets of keywords: “lumbar” or “spine”, “mechanical testing” or “*in vitro*” or “robotics” or “experimental”, “follower load” or “kinematics” or “load control” or “displacement load”. The wide and almost generic selection of keywords was necessary to avoid unwanted exclusions due to the high variability of terms found in spinal biomechanics field. All original papers between 1992 and 2022 were investigated. These results were filtered by excluding *in vivo* and clinical studies and those involving only *in silico* models. Only data from human or synthetic specimen were included. Particularly, we consider only experimental articles reporting the behaviour of lumbar single functional spinal units (FSU) or lumbar multi-level segment, also accepting the adjacent vertebrae T12 and S1. Finally, references of the selected articles were further reviewed and a complementary search was carried out. Data were extracted from text, tables and figures recurring to 'Graph Grabber' tool when necessary.

The experimental analysis of the mechanical behavior of the lumbar spine has been performed in literature with various set-ups differing for constraints, applied loads, and instrumentation. In the following, a synthetic overview is provided.

### Constraints

All the loading set-ups are fully constrained at the caudal vertebra, whereas the loads are transmitted to the cranial one which can be free to move (6 Degrees Of Freedom, DOF) or constrained within the sagittal plane (3 DOF). In the latter case vertebral coupled motions are not allowed and could over-estimate the spinal segment load response. In any case, all the studies evaluate the motion occurring on the sagittal plane.

### Instrumentation

Experiments were carried on in load-control or in displacement-control with load limits. The position of the load cell has not always been reported: most authors instrumented the most caudal vertebra^9,10,16,17,27^, but some other authors chose to instrument the most cranial one^13,19^. This detailed information can be relevant whenever experimental set-ups include a force which would result in a varying moment along the specimen axis: as demonstrated in the following, the moment produced by a force applied on the cranial vertebra cannot be well defined due to geometrical non-linearities resulting from large displacements taking place during loading.

### Applied Loads

The main pure applied load is a bending moment (M) whose value differs among various authors, ranging between 0.5 Nm^13^—2 Nm^1,2^ and 10 Nm^7,16,18,23,24,31^; most often it is equal to 7—8 Nm^5,9,11,17,25–27,31,32,34^, therefore 7.5 Nm value was used as a reference in the current work. A few authors apply different moments when working for extension or flexion: 8 Nm flexion coupled to 6 Nm can be found for example in Fielding *et al.* and Renner *et al.*^11,26^; one single work, finalized to the validation of dummies for crash tests, reached 182 Nm^9^.

As an addition to bending moment, a longitudinal load (LL) might be applied, with the aim of simulating the weight of the trunk. The value of this load is very variable in literature where 100–110 N^23,24^, 200–400 N^5,8,18^ and 500 N^16^ can be found. The direction of this load can be either always vertical (M + LL1), or can maintain the same relative orientation with the most cranial vertebra during the motion (M + LL2), or can be defined so that the force acts along the line connecting the cranial vertebra’s center to the caudal vertebra’s center (M + LL3). Configurations M + LL1 and M + LL2 are actually equivalent to vertical eccentric forces generating both a bending moment and a compression force^6,19^. Rarely bending moments were obtained by applying a shear force along the construct’s antero-posterior direction^9^.

Finally, many authors considered the so-called “follower load” which is an internal load pre-stressing vertebra-to-vertebra, due to muscular action. This load actually changes its direction when moving from one functional spine unit to the next one (M + FL). The follower load results in a sort of ‘locking’ between vertebrae and so it proved to be essential in order to provide sufficient stability when applying the highest bending moment (above 2–4 Nm). The follower load varies significantly from author to author, moving from 100 N^1,2^, through 280 N^27^ and 400 N^5,11,18,25^, up to 1200 N^16,25^. A simplified approximation of the follower load can also be obtained applying a force acting along the line connecting the cranial vertebra’s center to the caudal vertebra’s center. A more complex loading set-up was implemented by Wilke *et al.*^33^ who simulated specific muscular actions set equal to 80 N per vector pair.

Table 1 provides an overview of the experimental studies reviewed with the proposed literature search. Among the twenty-nine different articles found, 60% tested the spinal segment under a pure moment, while a longitudinal load was applied in 40% works. All the studies implementing a follower load fall within 1999 and 2013.

**Table 1:** Overview of the *in vitro* studies presenting experimental data on human or synthetic lumbar segment for flexion–extension (F–E) loads since 1992.

Study	Spinal levels (No. of samples)	Cranial vertebrae DOF	Load	Preload	Classification
Belwadi *et al.* (2008) [3]	T12–L2 (10), L4–S1 (9)	Sagittal motion	Force up to spinal failure	–	HLsf, M
Bennett *et al.* (2013) [4]	L4–L5 (6)	Free	8 Nm	FL 400 N	M, M + LL2
Bennett *et al.* (2015) [5]	L4–-L5 (6)	Free	8 Nm	FL 400 N	M, M + LL1, M + LL2
Borrelli *et al.* (2021) [6]	T12–S1 (1)—synthethic	Free	3 Nm (F); 2 Nm (E)	LL 1 N	M + LL1
Charriere *et al.* (2006) [7]	L5–S1 (7)	Free/Sagittal motion	2.5, 5, 7.5, 10 Nm	–	M
Cripton *et al.* (2000) [8]	L1–L2 (1), L2–L3 (3), L3–L4 (1), L4–L5 (1)	Free/Sagittal motion	5 Nm	LL 200, 400 N	M, M + LL1, M + LL2
Demetropoulos *et al.* (1998) [9]	T12–L5 (10)	Free	Cranial vertebra motion	–	HLsf
Di Angelo *et al.* (2019) [10]	T12–S1 (1)—synthetic	Sagittal motion and medium-lateral displacement	25° (F); 10° (E)	LL 20 N	M + LL3
Fielding (2013) [11]	L1–S1 (5)	Free	8 Nm (F); 6 Nm (E)	FL 400 N	M, M + FL
Gardner-Morse *et al.* (2004) [12]	L2–L3 (4), L4–L5 (4)	Free	1°	LL 0, 250, 500 N	M, M + LL3
Guo *et al.* (2016) [14]	L4–L5 (12)	Free	6 Nm	LL 400 N	M + LL1
Guan *et al.* (2007) [13]	T12–S1 (10)	Sagittal motion	0.5, 1.5, 2.5, 3.5, 4 Nm	–	M
Haher *et al.* (1994) [15]	T11–S2 (10)	Free	Eccentric force	–	M + LL1
Heuer *et al.* (2007) [16]	L4–L5 (8)	Free	1, 2.5, 5, 7.5, 10 Nm	–	M
Kelly *et al.* (2013) [17]	L1–S1 (2), L4–L5 (2)	Free	8 Nm	–	M
Kiapour *et al.* (2012) [18]	L1–S (8)	Sagittal motion	10 Nm	LL 800 N	M, M + LL3
Marras *et al.* (2021) [19]	T12–L3 (3), L1–L5 (1), L3–S1 (1)	Free	Eccentric force	–	M + LL1
Ou *et al.* (2021) [20]	T12–S1 (15)	Sagittal motion	Eccentric force	20% max. moment	M + LL1
Oxland *et al.* (1992) [22]	L1–S1 (5), L2–S1 (4)	Free	5 Nm	–	M
Panjabi *et al.* (1994) [23]	L1–S1 (5), L2–S1 (4)	Free	1, 2.5, 5, 7.5, 10 Nm	LL 100 N	M + LL3
Patwardhan *et al.* (1999) [24]	L1–S1 (5)	Free	10 Nm	LL 110 N, FL 1200 N	M + LL1, M + FL
Patwardhan *et al.* (2003) [25]	L1–S1 (21)	Free	8 Nm (F); 6 Nm (E)	FL 200, 400, 800, 1200 N	M + FL
Renner *et al.* (2007) [26]	L1–S1 (10)	Free	8 Nm (F); 6 Nm (E)	FL 800, 1200 N	M, M + FL
Rohlmann *et al.* (2001) [27]	L1–S1 (10)	Free	3.5, 7.5 Nm	FL 280 N	M, M + FL
Vergari *et al.* (2021) [28]	L1–S1 (3)	Free	8 Nm	–	M
Wang *et al.* (2014) [31]	L3–L4 (3)—synthetic	Free	7.5 Nm	–	M
Widmer *et al.* (2020) [32]	T12–L1 (6), L1–L2 (7), L2–L3 (3), L3–L4 (9), L4–L5 (7)	Sagittal motion and medium-lateral displacement	7.5 Nm	–	M
Wilke *et al.* (1994) [33]	L2–S1 (1)	Free	7.5 Nm	–	M
Zirbel *et al.* (2013) [36]	L1–L2 and L2–L3 (7), L3–L4 and L4–L5 (8), L5–S1 (6)	Free	7.5 Nm	FL 440 N	M + FL

Preload column: *LL* longitudinal load, *FL* follower load

## *In Silico* Meta-Analysis

### Multibody Model of the Lumbar Segment

A multibody model of the lumbar segment (L1–S1) was developed by Adams software (v. 2017, MSC Software, Hexagon Corporate Services Ltd., UK) in order to test the effect of different experimental loading conditions on the ROM’s magnitude. Geometries of the considered bones were derived by a commercially available anatomical model (SKU3430, Sawbones Europe AB, Malmö, Sweden) and the whole segment was aligned in space such that the L3 inferior endplate lied horizontally^34^. Each intervertebral disc was modelled as a revolute joint (1 degree of freedom) allowing the flexion–extension movement of two consecutive bone segments. The rotational axis of the joint is perpendicularly oriented to the sagittal plane and located at the midpoint of the line connecting the centroids of two adjacent endplates.

The relationship between reaction moment and flexion–extension angle for each FSU was assigned by using the following function^35^:

1 $$M\left(\theta \right)={C}_{3}{\theta }^{3}+{C}_{2}{\theta }^{2}+{C}_{1}\theta +{C}_{\mathrm{f}}F\theta$$

where *M* is the reaction moment, *θ* is the relative angle between two adjacent vertebrae with respect to their neutral position, *C*_i_ are coefficients specific for each disc, *F* is the compressive force acting on the intervertebral joint when the follower load contribution is considered, otherwise *F* = 0. The used function describes the global behaviour of the FSU considering also the action performed by constraining anatomical elements, such as ligaments and facet joints.

**Figure 1:**
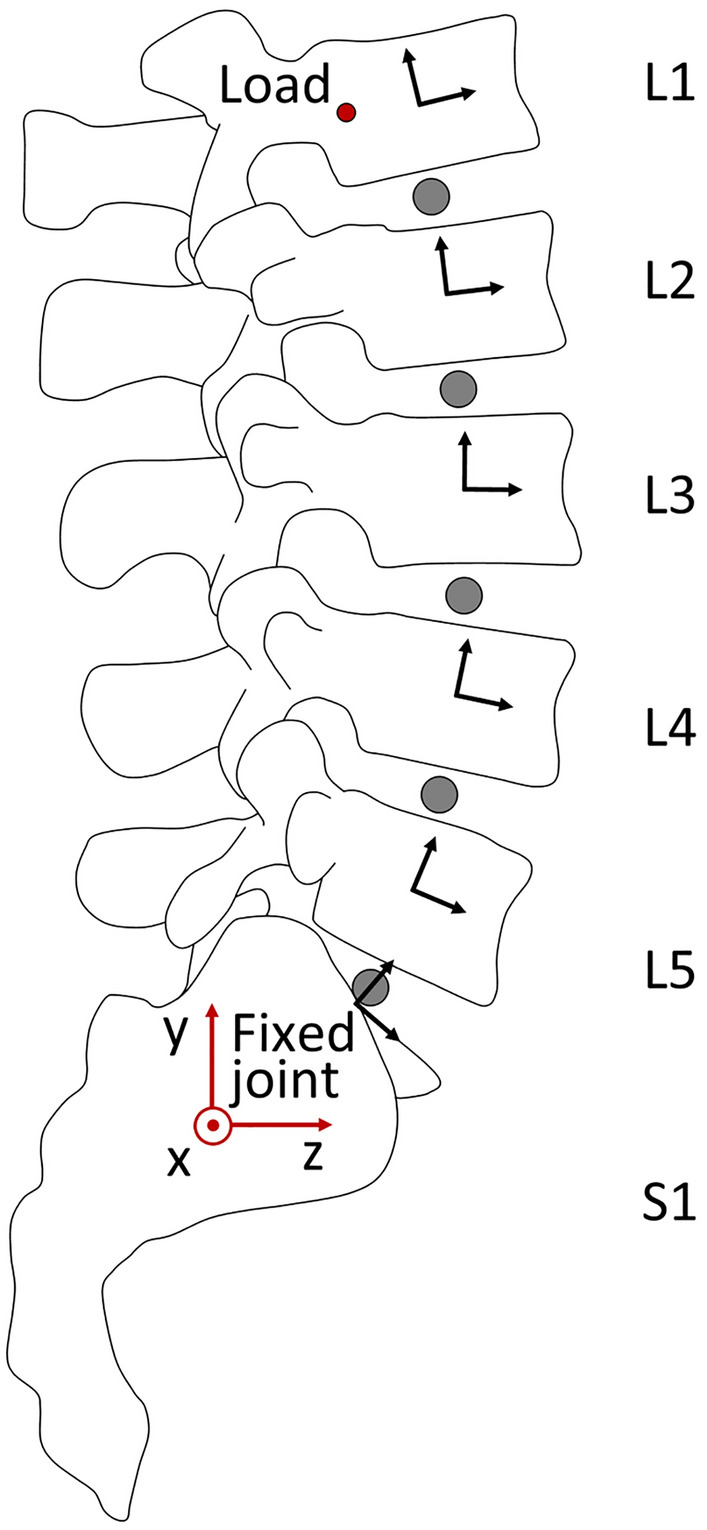
Lateral view of the multibody model of the lumbar segment.

The kinematics of the lumbar segment on the sagittal plane was described by defining a local reference frame at the centroid of each vertebral body. In particular (Fig. 1), the first axis of the local frame is parallel to the inferior endplate of the vertebra and points anteriorly, the second axis is perpendicular to the inferior endplate and points cranially, finally, the third axis results perpendicular to the sagittal plane and oriented to obtain a right-handed triad. Similarly, a global reference frame was also defined and located at the base of sacrum.

The final spatial configurations of the lumbar segment resulting from the different loading conditions were compared. In detail, the relative angles of rotation between adjacent vertebrae were measured and summed to obtain the total flexion and extension ROM. Furthermore, the applied moment generated at the S1 fixed joint and at each FSU were measured.

All the information required to replicate the multibody model are provided in the Supplementary Material section.

### Loading Conditions

**Figure 2:**
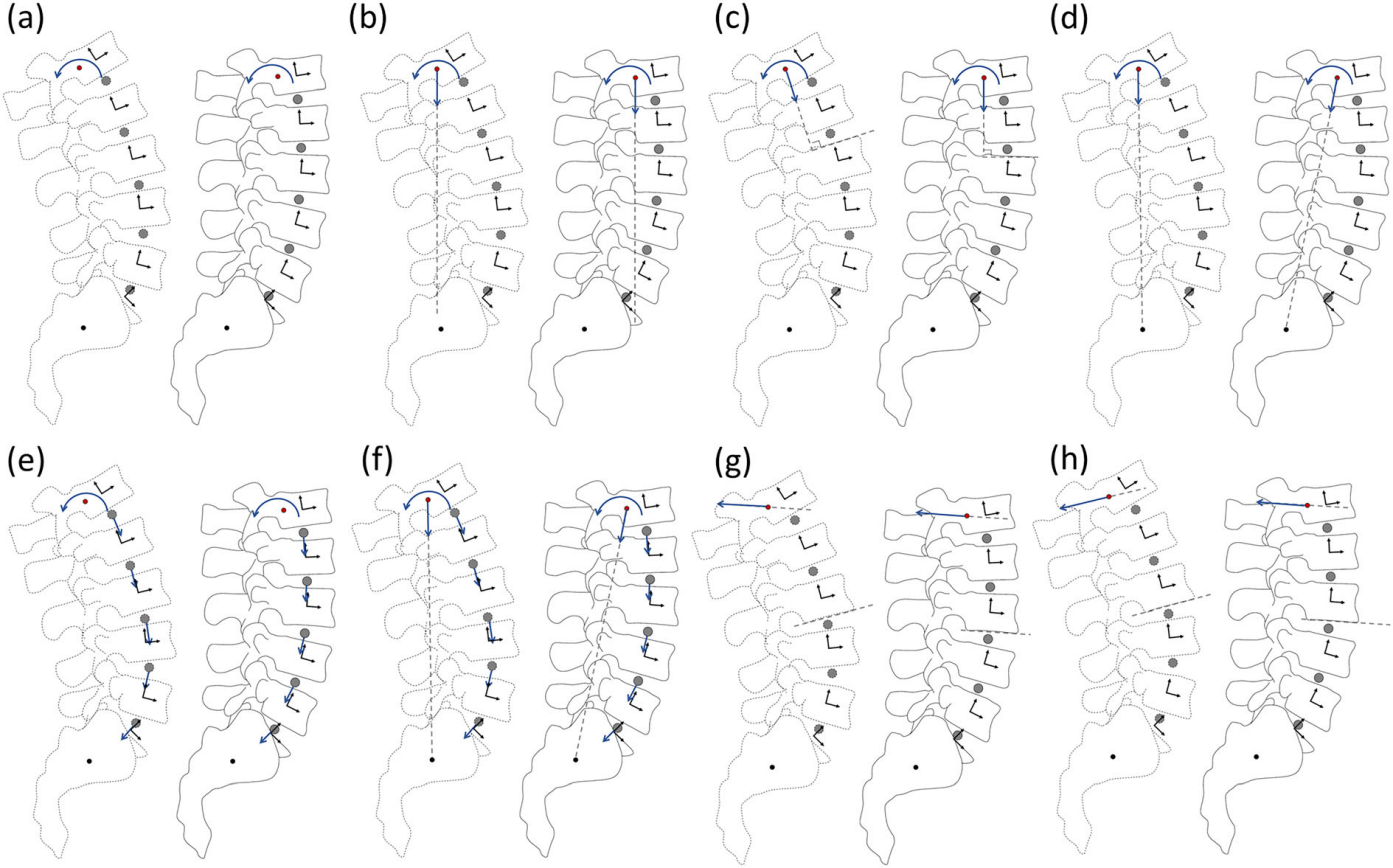
Loading conditions: (a) pure moment (M); (b–d) pure moment and longitudinal load (M + LL1, M + LL2, M + LL3); (e) pure moment and follower load (M + FL); (f) pure moment, follower load and longitudinal load (M + LL1 + FL); (f) pure moment and horizontal load (HLsf); (g) pure moment and moving load (HLbf). Solid line spine model represents the initial configuration; dashed line spine model represents the deformed configuration.

A total of eight different loading cases are here investigated, as summarized in Table 2 and illustrated in Fig. 2.

**Table 2:** Schematic representation of loading conditions and boundary conditions details.

	M				M + LL1				
			Fixed	Moving				Fixed	Moving
	M [Nm]	7.5		–		M [Nm]	7.5		-
	F [N]	–	–	–		F [N]	100		–
	FL [N]	–	–	–		FL [N]	–	–	–
	M + LL2				M + LL3				
			Fixed	Moving				Fixed	Moving
	M [Nm]	7.5		–		M [Nm]	7.5		–
	F [N]	100	–			F [N]	100		–
	FL [N]	–	–	–		FL [N]	–	–	–
	M + FL100–M + FL280				M + LL3 + FL280				
			Fixed	Moving				Fixed	Moving
	M [Nm]	7.5		–		M [Nm]	7.5		–
	F [N]	–	–	–		F [N]	100		–
	FL [N]	100 280	–			FL [N]	280	–	
	HLsf				HLbf				
			Fixed	Moving				Fixed	Moving
	M [Nm]	7.5/L*		–		M [Nm]	7.5/L*	–	
	F [N]	–	–	–		F [N]	–	–	–
	FL [N]	–	–	–		FL [N]	–	–	–

L* spine length

The loading cases were taken from literature and are described in the following; the respective references can be found in Table 1: firstly, a pure flexion–extension moment load (M) of 7.5 Nm was applied to the centroid of L1 (Fig. 2a). Then the pure moment was combined with three different forms of compressive longitudinal pre-loads, all sharing the same magnitude equal to 100 N. In the first load condition (M + LL1, Fig. 2b), the vertical load is oriented along the longitudinal axis of the spine at rest and it is fixed in space throughout the testing. The load condition M + LL2 (Fig. 2c) is realized by a compressive load which is initially oriented along the longitudinal axis and, during the motion, maintains a fixed orientation with reference to the cranial vertebra. Finally, the pure moment was combined with a compressive load (Fig. 2d) directed from the centroid of L1 to the S1 fixed joint location (M + LL3). Other loading cases (Fig. 2e) consist of the pure moment combined with two different values of follower load, here simulated equal to 100 N (M + FL100) or 280 N (M + FL280), respectively; the configuration M + LL3 + FL280 consists of a combination of the pure moment with the 280 N follower load and load LL3 (Fig. 2f). The last two load conditions investigated (Fig. 2g and 2h) correspond to a horizontal force of 41.7 N (i.e., 7.5 Nm divided by the spine length L*) applied to the centroid of L1, having its orientation fixed in space (HLsf) or moving with the L1 vertebra (HLbf). Generally, the pure moment and/or loads were applied on L1 while the S1 was fully constrained. Whenever moments measured at the most cranial vertebra and at the most caudal one were not equal, the analysis was repeated, assuming the positioning of the load cell at the sacrum and limiting the respective reacting moment to 7.5 Nm. To do that, the secondary longitudinal load was applied first, while the bending moment was incrementally applied according to a ramp as far as the limit value 7.5 Nm was reached. In this configuration, the sagittal torque component records not only the pure moment but, eventually, also spurious moments generated by compressive loads (e.g., M + LL1).

In this study, the pure moment loading condition was considered as a reference.

## Results

The following section illustrates the results of the numerical lumbosacral spine model with the scope of shedding light on the effects of boundary loads only and providing the reader with a tool to critically compare the *in vitro* experimental results.

The multibody model here implemented to be used as benchmark (M) was validated against ROM of the various FSUs from L1 to S1, as reported in experimental studies in the literature^13,23^; details regarding this validation are reported in the Online Appendix (Supplementary Information section, Fig. A1).

**Figure 3:**
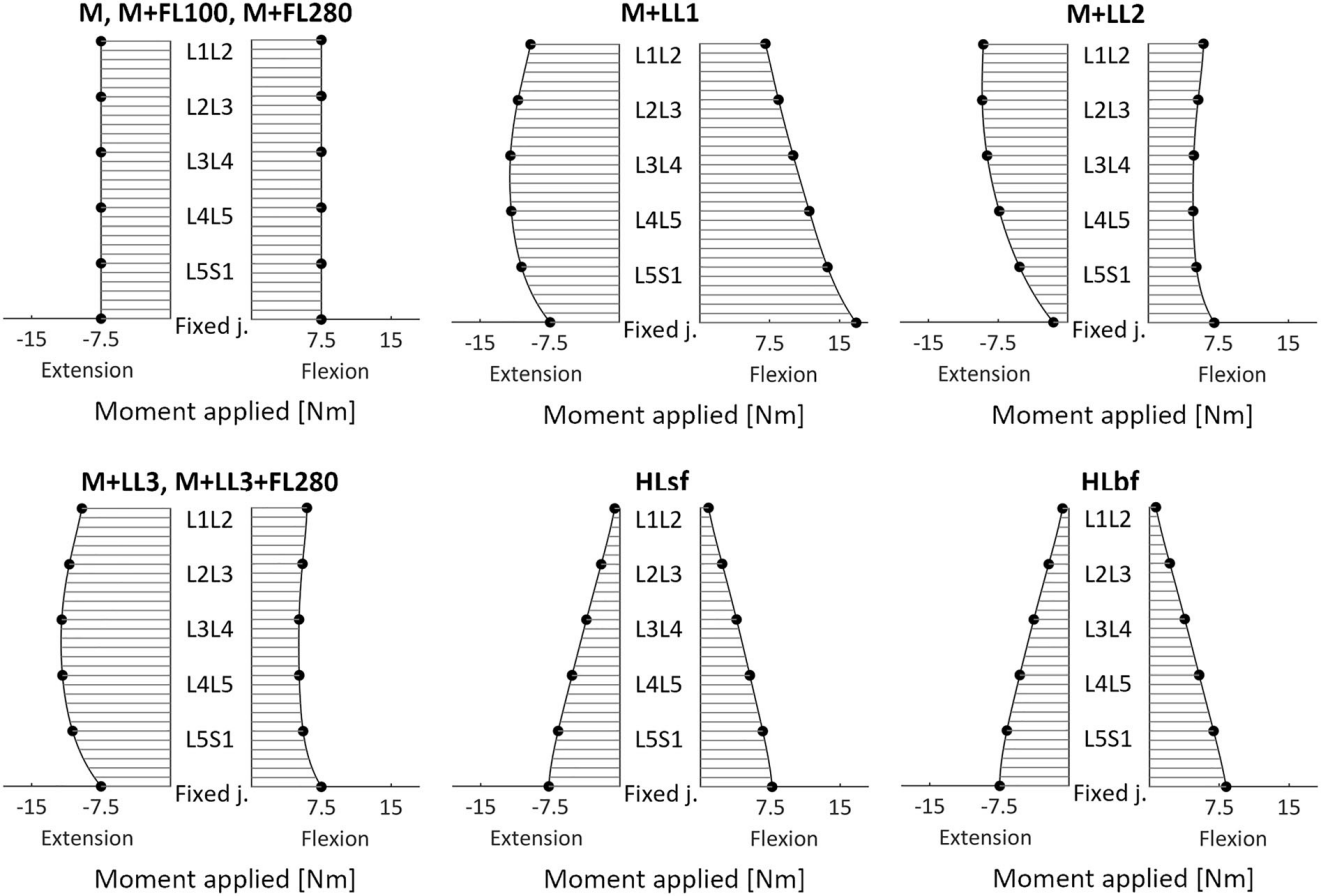
The moment diagram shows the moment measured at the intervertebral joints (L1L2–L5S1) and at the fixed joint.

Moment diagrams in Fig. 3 show how the moment is actually transferred along the spine, once the defined loads are applied both in flexion and extension. Load conditions which resulted in the same diagram were unified. As expected, in the case of the pure moment, the load along the segment is constant and the moment applied to the uppermost vertebrae is equal to the load at the caudal fixed joint. Conversely, the application of a second load creates non-uniform deviation along the vertebral levels, according to geometric non linearities. More in detail, all the longitudinal loads produce non-linear moment trends. Regarding M + LL1, during posterior bending the lordotic curve increases: therefore, the vertical compressive force has a non-uniform arm with respect to the vertebrae, reaching its greatest value at L3L4 joint (11.7 Nm). This causes an additional moment load contribution resulting in a greater rotation of the vertebrae as shown in Fig. 3. The moment growth ranges from + 27.6% at L1L2 to more than 55% at L3L4 and L4L5. Moreover, it is important to note how the application of this vertical load (M + LL1) affects the resulting moment at the fixed caudal joint. In extension, it initially generates an opposite flexion moment due to the anterior position of L1 with respect to the sacrum. During the motion, the location of the force moves behind the joint, contributing to extension. This setting causes a non-univocal correspondence between the applied moment (measured by a top load cell) and the reacted moment (measured by a bottom load cell). However, in the model here described, the direction of the vertical force passes close to the fixed joint for 7.5 Nm applied moment, therefore this aspect is not evident. The analysis was therefore repeated at − 5 Nm and − 10 Nm: for these loading conditions, the resulting reaction moments were − 44.4 Nm (− 11.1%) and − 10.4 Nm, (+ 4%), respectively. Conversely, looking at the anterior bending a flexion moment of 7.5 Nm applied by the “testing machine” at L1 is more than doubled at the fixed joint (16.8 Nm, + 124%). By orienting the compressive load accordingly to L1 motion (M + LL2), the moment diagram assumes another shape. In extension, the resulting moment along the lumbar joints decreases monotonically with a maximum, minimum and mean variation along the lumbar vertebrae, with respect to the pure moment, equal to + 22.8%, − 1.7% and 15.56% respectively, and reaching remarkable deviations from pure moment load at the lumbosacral joint (− 30.8%) and the fixed joint (− 79.5%). In flexion, LL2 is oriented posteriorly to the intervertebral joints providing an extension moment which reduces the loads and consequently the rotation of the vertebrae; this effect can be almost considered constant for all vertebrae (− 30.6 ± 5.9%). Finally, the M + LL3 load condition partly resembles to both previous longitudinal load cases: on the one hand, in extension, the diagram differs from M + LL1 by less than 4%, on the other hand, in flexion the diagram differs from M + LL3 by less than 8%.

The moment diagram related to the horizontal loads (HLsf, HLbf) can be straightforwardly associated to a cantilever beam subjected to a perpendicular force at one extremity. The moment at the joints increases almost linearly from L1L2 to L5S1 with a deviation from pure moment equal to − 91% and to − 8%, respectively.

The loading conditions including the follower load return the same moment diagram as the pure moment, independently from its magnitude, since its main effect is the variation of the coefficients in the resistant moment-rotation law of revolute joints (Eq. 1).

**Figure 4:**
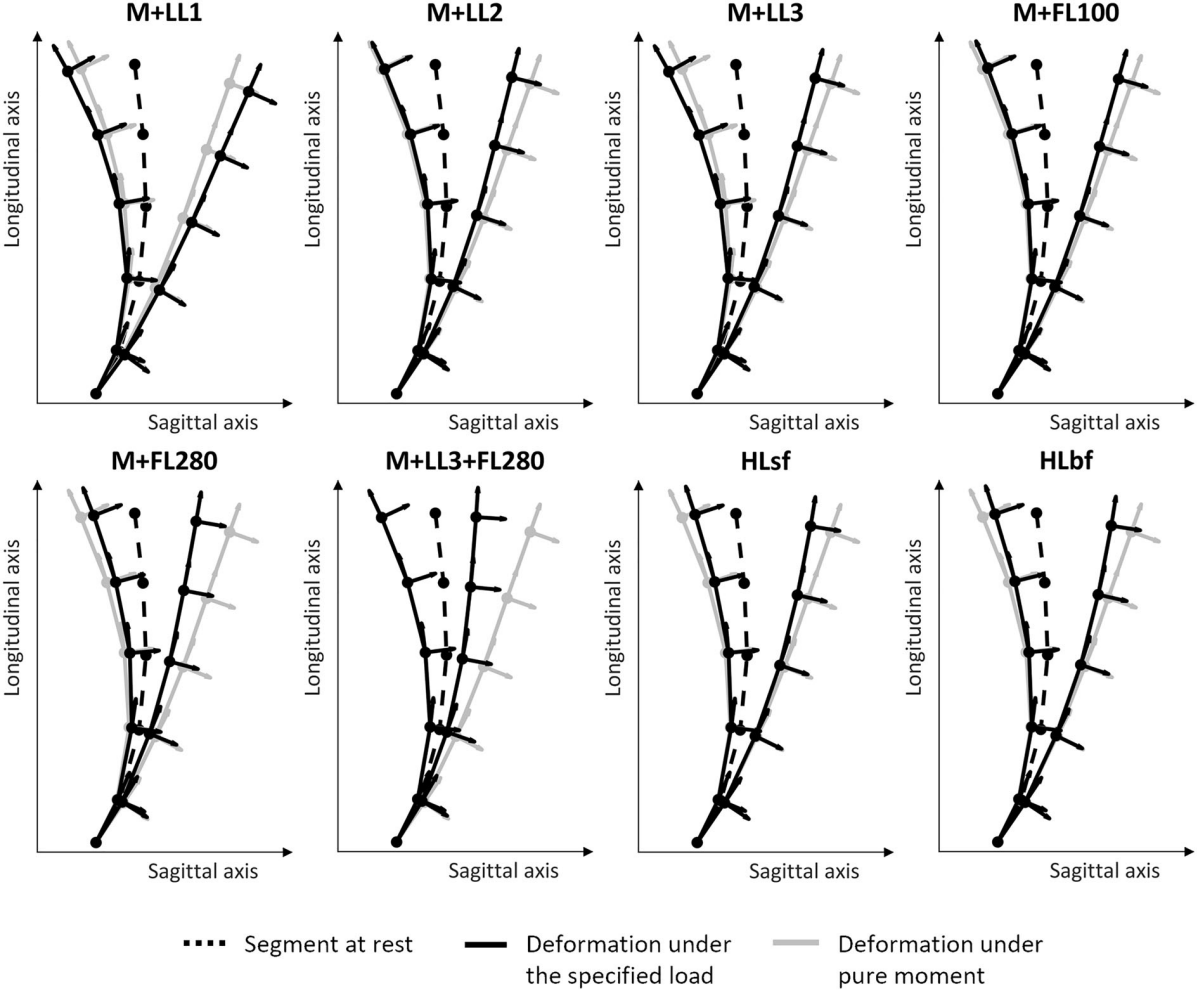
Deformation of the benchmark multibody model in accordance to the applied loads (black) both in flexion (to the right of the dashed rest line) and in extension (to the left of the dashed rest line). The grey deformation corresponds to the 7.5 Nm pure moment.

For each loading condition, Fig. 4 shows the deformation of the lumbosacral segment along the sagittal plane at the maximum of anterior and posterior bending; the ball-and-stick representation depicts the centres of mass of each vertebral body (Fig. 1, L1–S1) linked by a straight line. For each vertebral level, the local orientation is represented.

In flexion, all different loading conditions reveal a stiffer behaviour than the M model, exception made for the M + LL1 case. Concerning the extension behaviour, the addition of a longitudinal pre-load increases the motion of the segment in LL1 and LL3 cases, while LL2 appears not to change the global deformation. Conversely, the “follower load” reduces the mobility of the segment increasingly for higher magnitudes of the pre-load. The application of a shear horizontal force results in a reduced bending both in extension and in flexion.

**Figure 5:**
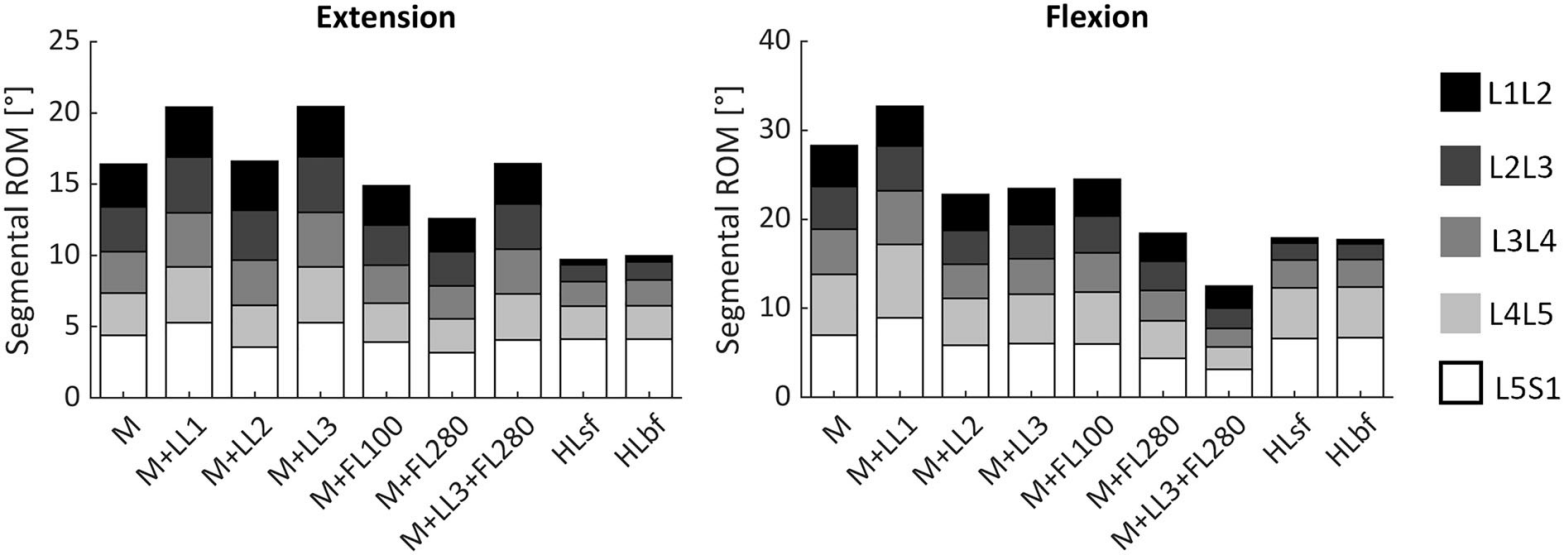
Segmental range of motion (ROM) in extension and flexion for each loading case.

The total ROM of the lumbosacral segment and how it is shared among segmental levels, is further showed in Fig. 5. Table 3 gives details about the total ROM difference for each loading condition, considering flexion and extension both separately or merged together as a unique sagittal ROM.

**Table 3:** ROM difference for each loading condition with reference to gold standard pure moment

	Extension	Flexion	Flexion–Extension
M + LL1	+ 24.6%	+ 15.5%	+ 18.9%
M + LL2	+ 1.26%	− 19.6%	− 12.0%
M + LL3	+ 24.7%	− 17.1%	− 1.8%
M + FL100	− 9.1%	− 13.6%	− 11.9%
M + FL280	− 23.1%	− 34.8%	− 30.5%
M + LL3 + FL280	+ 0.2%	− 55.8%	− 35.3%
HLsf	− 40.8%	− 36.7%	− 38.2%
HLbf	− 39.2%	− 37.4%	− 38.0%

The comparison is computed evaluating only extension, only flexion, and summing both contribution in one unique total term

Segmental motion induced by horizontal loads (HLsf, HLbf), are very different from a pure moment: in both bending sides the segmental motion increases cranio-caudally with an almost null contribution at the L1L2 (< 1°) level and a L4L5 and L5S1 which cover almost the 70% of the total ROM.

The LL1 pre-load increases the total ROM in both directions, and this happens at all segmental levels, exception made for L1L2 in flexion (− 4%). In extension, the levels which more differ from the pure moment are the middle ones (L3L4 + 31.7%, L4L5 31.2%), whereas in flexion the caudal levels are the ones undergoing the greatest change of ROM (L4L5 + 21.1%, L5S1 + 28.1%). Interestingly, the LL2 pre-load doesn’t vary the total ROM in extension at 7.5 Nm but the distribution of the single segmental ROM is slightly remodeled: the contribution of L5S1 on the total ROM decreases of 5% (from the 26.8 to 21.33% of the corresponding total ROM) and this difference is equally partitioned among the first three cranial levels. In flexion, the model subjected to LL2 or LL3 shows a drop of the total ROM almost homogeneous among all segment (− 19.6 ± 4.3% and − 17.2 ± 3.7%, respectively). In the case of LL3 it is worth mentioning that, despite this load doesn’t generate any moment at the fixed joint, it makes roto-translate all single vertebrae, leading to two opposite behaviours: an increment of the segmental ROM in extension, and a decrease in flexion compared to the pure moment (Table 3). A remarkable aspect is that those differences are counter balanced in the flexion–extension ROM and, without separating the motion, that information would be lost.

To conclude, the addition of the follower load enregisters a stiffening of the model causing a reduction of the total ROM proportional to the magnitude of the pre-load.

In addition to that, we separately further analysed the case of flexion in M + LL1 and extension in M + LL2. As previously described, those cases have in common a significant deviation between the moment applied at the cranial vertebra and the resultant moment at the fixed joint. Thus, we evaluated the segmental ROM when 7.5 Nm was measured at a load-cell located on the caudal vertebra: with reference to M + LL1, the resultant moment would derive mainly from the force component rather than from the applied moment, which ultimately would be limited to 1.8 Nm. Consequently, the ROM decreases up to 22.5°, from + 15.5% to − 64.1% of the ROM related to the pure moment; the distribution of deformation among segments becomes extremely uneven with some components almost null (both L1L2 and L2L3 < 1°) and the lumbosacral joint plays s major influence, covering more than 50% of the total ROM. Conversely, in M + LL2 case, as expected, the load cell set at the fixed joint made the motion of the model extended, with the total ROM incremented of 7.1°, and the single vertebral levels increased uniformly (+ 1.4 ± 0.3°). Nevertheless, due to the counter moment of LL2, the moment applied almost doubled (14.4 Nm) as well as the moment at the single intervertebral joints (max. 16 Nm at L2L3, min. 11.3 Nm at L5S1).

## Discussion

The *in silico* benchmark was aimed to shed lights on the importance of setting up proper boundary conditions in order to avoid introducing biases and to guarantee the best reproducibility of experimental tests, for the sake of comparison of results. Previous authors, have already outlined the impact of over constraining: Walker *et al.*^30^ who worked on porcine vertebrae showed how 10 Nm bending moment in a 3 DOF system (that is with all three translations prevented) might results in additional 100 N axial load and 50 Nm torsional load. The modalities of application of a longitudinal force can be even more critical and there is no uniformity in loading application techniques in literature as well as in the portion of tested lumbar spine; this aspect makes it difficult to carry on a rigorous comparison between the numerical model here proposed and these works, nevertheless general trends related to differences in boundary conditions could be inferred.

According to results, the addition of a longitudinal load can produce different effects on the spine ROM, based on the load application procedure (LL1, LL2 and LL3): in these scenarios, artefacts related to the pre-load application (Fig. 3) arise, resulting in an amplification of the applied moment.

Different effects of the longitudinal load configuration have been experimentally investigated in the work of Cripton *et al.*^8^ for single FSUs, focusing on ROM’s variations and quantifying the amount of artefact moments. Numerical outcomes for the LL1 load configuration can be straightforwardly extended from single FSU results to multiple FSUs^8^: Cripton *et al.* have assessed an increment of spine flexibility for a pre-load with fixed vertical orientation, as here observed in the numerical spine model (Figs. 1, 4). This greater flexibility is apparent, and it is due to a higher applied moment, as a consequence of artefact moments (Fig. 3)^29^. Cripton *et al.*^8^ also performed statistical analyses in order to assess significance of these results with respect to the case of no pre-load application: even if experimental tests did result in greater ROMs both in flexion and extension, those variations did not prove to be statistically significant due to specimens’ biologic variability; this limitation was here overcome thanks to the use of a numerical model. Referring to the additional moment induced by the application of the vertical load, ROM increments ranging between 15 and 40% have been found by Cripton *et al.* for a pre-load of 200 N; this trend is in agreement with the one observed in numerical results (Fig. 2). It should be here stressed that the highest artefact moments (+ 56.7% in flexion and + 55.3% in extension) are reached at the central vertebral in the case of a 100 N pre-load, due to the respective peak moment arm with reference to the load applied on L1. Whenever additional loads are applied at the cranial vertebra, the spine curvature plays a major influence, resulting in additional moments which are different from vertebra to vertebra and which may change at different loads as a result of geometric non-linearities. Due to the major experimental efforts required by including various consecutive FSUs in mechanical tests, a limited number of authors^10,13,17^ have investigated the flexibility of large portions of lumbar spine: in order to be able to validate spine numerical models for different loading conditions and reach more definite conclusions, further tests of this kind are encouraged.

For what concerns the effects of the follower load, the same work by Cripton *et al.*^8^ tested a single FSU with a pre-load (200–400 N) passing through the intervertebral disk’s center. The flexion–extension ROM underwent only slight increases in this case since no additional artefact moments occurred. Other works have considered the full lumbar spine segment, assessing the effects on spine flexibility of the follower load with respect to the pure moment condition. In the works of Rohlman *et al.*^27^, Patwardhan *et al.*^25^ and Renner *et al.*^26^ different levels of pre-load (280 N, 0–1200 N, 800 N respectively) have been investigated, using similar experimental set-ups. According to numerical results here obtained, the follower-load results in a stiffening effect, with a resulting total flexion–extension ROM reduction equal to about − 30.5% for a 280 N load; moreover, a decreasing trend for the ROM was associated to an increasing value of the applied follower-load. The same behaviour can be observed in experimental tests, with ROM’s reduction equal to − 22% with a 800 N pre-load^26^ and up to − 25.4% for a 1200 N load^25^. In addition, all these works agree that the more the follower load magnitude increases, more increments the load-carrying capacity of the segment. The numerical model here presented seems to provide a greater stiffening effect than those reported in these experimental studies, considering that a maximum load of 280 N was here implemented and Patwardhan *et al.* stated that they found significant differences from the pure moment condition only for follower loads greater than 400 N. The reason for this might be that the designed multibody test benchmark is indeed affected by some major limitations: firstly, the mechanical behavior of the intervertebral disk has been simulated based on an empirical non-linear law of revolute joints^35^. In addition, the interlocking action produced by the follower load was taken into account including its action in the analytic formulation; however, vertebra-to-vertebra pre-compression and relative displacements due to facet engagements^5,16^ could not be simulated. Consequently, the centers of rotation remain fixed during spine flexion/extension: this is not a faithful reproduction of the actual spine behavior, as witnessed by experimental set-ups where the follower load was implemented. In facts, this load was simulated through two cable wires, constrained superiorly and inferiorly attached to an actuator or to a vertical dead weight. These wires passed through eyelets (or, generally, guides) fixed at the center of both lateral sides of the vertebral bodies in order to follow the spine curvature. The hypothesis which stands at the basis of this set-up is that the wires intercept the axis of instantaneous rotation and do not produce any spurious moment or shear force. However, a continuous adjustment of the follower load path was required as the spine bent due to variation of the location of vertebra-to-vertebra instantaneous center of rotation^24^.

The analysis of various set-ups was made somehow hard due to incompleteness of information provided by authors: more in detail, few authors report the position of the load cell and of the controlled pure moment, and this could not be indifferent due to the action of ‘spurious’ loads. As an addition the term ‘axial load’ might be rather vague since it is fundamental to know how this force is directed as the spine deforms.

The analysis here performed with reference to bending moment should be transferred to other main motions such as torsion and lateral bending; nevertheless, the model is expected to be adequate to provide insight into these additional movements.

### Supplementary Material

Supplementary material for the reproduction of the multibody model is available at https://data.mendeley.com/datasets/8jhb5bpxjm/1.

## Supplementary Information

Below is the link to the electronic supplementary material.

Supplementary file1 (PDF 295 kb)
