# Supplementary material for: In Silico Meta-Analysis of Boundary Conditions for Experimental Tests on the Lumbar Spine
Source: Ann Biomed Eng. 2022 Jul 29;50(10):1243–54. doi: 10.1007/s10439-022-03015-x (PMC9474587; doi:10.1007/s10439-022-03015-x)
Supplement: Supplementary file 1 — Supplementary file1 (PDF 295 kb) [file 10439_2022_3015_MOESM1_ESM.pdf]

APPENDIX

This section is dedicated to provide a brief insight regarding the behaviour of our model and proof its validity. A ramp moment was applied to the model up to 7.5 Nm; for each segmental level, the moment-ROM curves were compared with the state-of-the-art results (Fig. A.1). On the one hand, Guan et al.[13] revealed crucial to show the optimal correspondence of the model for small displacements: the model always fitted within the experimental results. On the other hand, Panjabi et al.[23] was used to evaluate the model at load larger than 4 Nm. Generally, Panjabi results are reported as less stiff than other experimental results[7,31,36], and this is clearly confirmed by the 2.5 Nm point in the figure, where both Guan et al. and Panjabi et al. results are available. Nevertheless, the model follows the same trend of experimental results and is found within one standard deviation except for L2L3 (the deviation can be still considered negligible at 7.5 Nm).

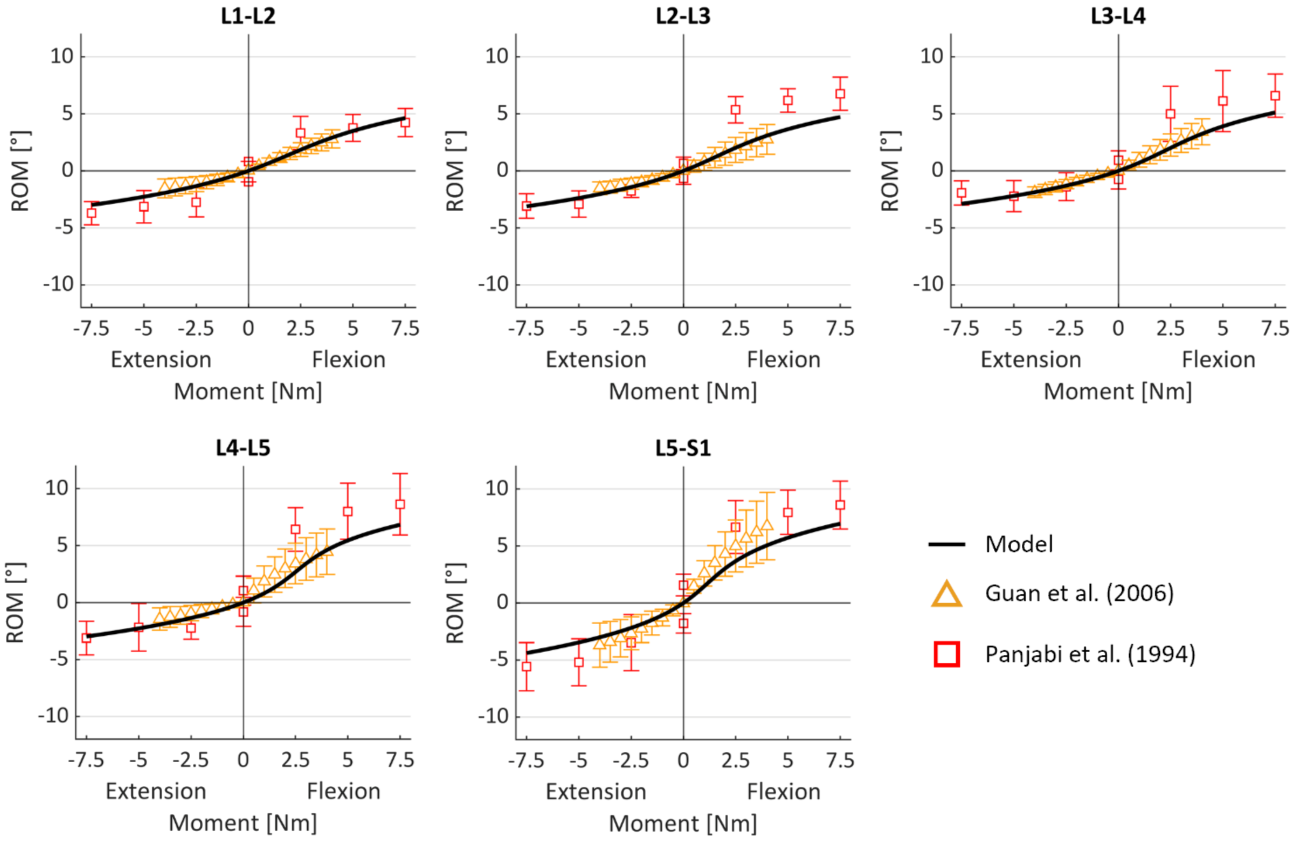

FIGURE A.1. Moment-rotation behaviour of each single level in flexion and extension, with applied moment of ±7.5 Nm at L1 and S1 constrained.
